# Effects of a web-based rehabilitation aftercare on subjective health, work ability and motivation: a partially randomized controlled trial

**DOI:** 10.1186/s12891-021-04239-z

**Published:** 2021-04-19

**Authors:** Darko Jekauc, Sabine Rayling, Sara Klopp, Detlef Schmidt, Lena-Marie Rittmann, Julian Fritsch

**Affiliations:** Institute for Sport and Sport Science, Karlsruhe Institute of Technology, Engler-Bunte-Ring 15, 76131 Karlsruhe, Germany

**Keywords:** Digital platform, Rehabilitation, Aftercare, Health, Work ability, Web-based, Motivation, Attendance

## Abstract

**Background:**

Rehabilitation is seen as crucial in dealing with the demographic change in many European countries. In Germany, for example, after having stayed in a rehabilitation center, patients have the possibility to participate in aftercare programs aimed at promoting long-term health behaviour. Despite the relevance of follow-up support for patients’ long-term health and work ability, participation rates in aftercare programs are quite low. Here, web-based aftercare programs can be a viable alternative to the traditional face-to-face programs due to their flexibility in time and location. This research project aims to use quantitative and qualitative methods to gain more insight into the potential of web-based aftercare programs.

**Methods:**

The goal is to recruit up to 1150 patients at baseline in five rehabilitation centers across Germany. For ethical reasons, partially randomized experimental study design is used to quantitatively assess the effectiveness of web-based aftercare programs. All patients are offered the traditional face-to-face aftercare treatment (IRENA). When patients deny to participate in traditional face-to-face aftercare, they are randomly distributed into either web-based aftercare (digIRENA) or a control group. In all three groups, the SF-12, which measures subjective health, and the WAI, which measures working ability, will be used at baseline, 13 weeks, 26 weeks and 43 weeks after the patients have left the rehabilitation center. BREQ-2, which measures motivation, is used only in the traditional aftercare group and the web-based aftercare group. A multivariate analysis of variance with repeated measurement and latent growth curve models will be used to compare the development of the variables in the three groups. For the qualitative part of the study, interviews with patients and therapists will be conducted to shed light on the applicability, acceptance, and usability of web-based aftercare programs.

**Discussion:**

This study may provide valuable insight into the potential of web-based rehabilitation aftercare programs as a way to supplement traditional face-to-face programs. This seems particularly promising if it can manage to reach those patients who do not currently participate in traditional face-to-face rehabilitation aftercare programs due to time and location constraints.

**Trial registration:**

The trial has been registered at the German Register of Clinical Studies (DRKS) with the registration number: DRKS00022467.

**Supplementary Information:**

The online version contains supplementary material available at 10.1186/s12891-021-04239-z.

## Background

The demographic change in European countries is associated with a shrinking and ageing of the population [1, 2]. These changes are associated with an increase of people receiving a pension, even though there are less people in work who can support this welfare system. This development points towards the importance of promoting actions that aim at improving the health of the working population [3].

One important asset in the promotion of health of the working population is the rehabilitation sector. The aim of rehabilitation is not only to support the recovery process, but also to help patients develop a healthier lifestyle. Studies show that a long-term effect of rehabilitation is difficult to achieve and depends largely on the follow-up support [e.g., [4, 5]]. For instance, in Germany patients have the possibility to participate in an intensive rehabilitation aftercare program after having left the rehabilitation center. Although the costs for this program are covered by the German pension insurance fund, less than 15% of the patients participate in aftercare programs [6]. There are several reasons that may explain this discrepancy between a program that promotes the long-term effects of rehabilitation and the low number of patients that take advantage of this possibility. One main reason is that it is difficult to find time after having returned to work to visit the facilities where aftercare programmes are offered, often during limited time slots. Furthermore, especially in remote areas, the distance between the place of residence and the nearest facility may be too big for regular participation.

The development of technology associated with the increasing possibility of web-based platforms may provide a promising alternative to traditional face-to-face aftercare programs. While new technologies open up possibilities for the entire health care system [7], the term telerehabilitation refers specifically to the delivery of various rehabilitation services via web-based technologies [8]. Web-based rehabilitation aftercare programs could help make health care services more efficient, more accessible and more affordable to larger parts of the population [9]. Irrespective of location and time, patients and therapists can communicate with each other via a web-based platform. The patients do not need to be physically present in the rehabilitation facility and can be supported by supplementary information material via the platform. This flexibility appears to be particularly promising in light of the low number of patients taking part in aftercare programs [10].

Web-based rehabilitation has been shown to be effective in the treatment of cardiovascular diseases [11], neurological applications [12] and in orthopaedic rehabilitation [8, 13]. In addition, empirical evidence suggests that web-based rehabilitation might be a cost-effective tool, compared to traditional rehabilitation [14].

Despite the evidence for clinical and cost effectiveness, web-based rehabilitation is still not widely disseminated [15]. Besides general scepticism about the dangers of the increasing influence of technologies, potentially bringing precarious employment conditions for medical staff [16], another reason may be the lack of studies focusing on more “soft” parameters such as work ability, subjective health, and motivation. The work ability is a result of factors that enable a person to successfully deal with their work demands [17]. A low work ability has been shown to predict the risk of long-term absence at work [18] as well as an early retirement [19]. Subjective health refers to how an individual perceives their own state of health, which might differ from objective health parameters [20]. Longitudinal studies have shown that the subjective health is a predictor of the use of health services [21], and also mortality [22]. Finally, considering the importance of a long-term behaviour change, a plethora of studies highlights that more autonomous types of motivation are associated with the adoption and maintenance of a healthier lifestyle [23].

**Fig. 1:**
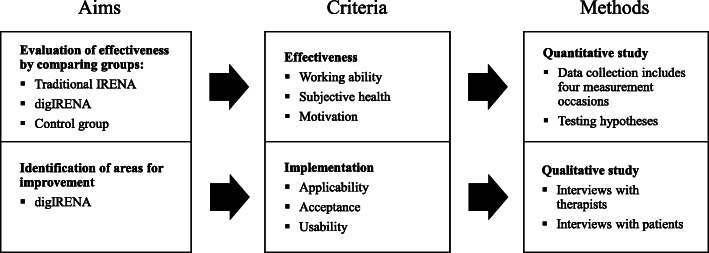
Overview of the aims, criteria, and methods of the digIRENA Study

To conclude, web-based aftercare programs appear to offer a promising alternative to traditional aftercare programs, which may help to ensure the long-term effects of rehabilitation. Using a mix-method design, the purposes of this current study are to assess the efficacy of a web-based platform used in the aftercare of a rehabilitation stay as well as to gain deeper insights into how a web-based platform is perceived by therapists and patients (see Fig. 1).

## Methods

In order to fulfill the purposes of the study, a quantitative and a qualitative study were conducted. An overview of the study is depicted in Fig. 1.

### Quantitative study

In order to examine the effectiveness of the web-based aftercare an intervention study is conducted, comparing a web-based aftercare program with traditional aftercare program and a control condition.

#### Intervention

In this study, two rehabilitation aftercare programs are implemented: the traditional face-to-face aftercare program (IRENA; Intensified Rehabilitation Aftercare) and the web-based aftercare program (digIRENA; digitized Intensified Rehabilitation Aftercare). Both aftercare programs consist of 24 sessions and are tailored to the orthopaedic conditions of the rehabilitants [24]. The program has to be conducted by a qualified physiotherapist who selects the contents of the aftercare program. In the web-based aftercare program, the subjects receive a comparable individualized program with the same number of sessions as the traditional face-to-face IRENA. The web-based program is supervised by an external team of physiotherapists, employed by the provider of the web-based platform. Moreover, the web-based platform provides tools such as relaxation exercises or cooking recipes equivalent to the multimodal approach of a traditional face-to-face aftercare program. In both programs, if patients do not attend a session for at least six subsequent weeks, they are considered dropouts. If inactive, the intervention provider in the web-based aftercare program can encourage the patient to remain in the intervention. Patients in both aftercare groups are continuously supervised by qualified therapists during the interventions. In case of possible harm from the intervention, patients can be referred to appropriate treatment. The participants in the control group receive neither IRENA nor digIRENA. However, they have the possibility to organize their own rehabilitation activities. Finally, all patients can receive additional therapies depending on their health status.

#### Sampling and participants

Patients with orthopedic complaints who have applied for and been approved for rehabilitation on the prescription of a primary care physician are recruited from five rehabilitation centers throughout Germany. The inclusion criteria are that patients are at least 18 years old, have a basic knowledge of the German language, and their primary diagnosis concerns an orthopaedic problem. Furthermore, only patients who are insured with the public pension insurance Knappschaft Bahn See can participate in the study, as the included rehabilitation centres belong to Knappschaft Bahn See. When patients are informed about the possibility to take part in an aftercare program during their stay, they are also asked to participate in the study. In case they give their consent, they are assigned to three groups: (1) traditional face-to-face aftercare program (IRENA), (2) web-based aftercare program (digIRENA), or a control group. For ethical reasons, a partial randomized controlled trial was used. All patients are first asked whether they want to take part in the traditional aftercare program (IRENA). Only if they deny, they are randomly assigned to the web-based aftercare program or the control group. This means that the IRENA condition was offered preferentially and the randomization referred only to the digIRENA and control condition. Sealed envelops are used for the randomization and patients only know their group after opening the envelop. The envelops are distributed with the goal to obtain a ratio of 1:1 between the two groups. Due to nature of the study neither participants nor staff can be blinded to allocation. As an incentive to take part in the study, all patients who complete the study with all measurement occasions receive a 50 Euro Amazon voucher.

Because only a small number of studies exists in relation to web-based aftercare interventions, we chose to take a conservative approach in the power analysis with a small estimated effect size [8]. Conservatively estimating the effect size by Cohen’s *f* = .15, *α* = .05, 1-*β* = .80, with three groups, a correlation among repeated measures of *r* = .30 and a design with three repeated measurement occasions, on average, using an ANOVA with repeated measures (within-between interaction), the calculated total sample size is 573 participants, nearly equally distributed over three groups. Since we expect a dropout rate of about 50% [25], the plan is to recruit 1150 patients.

#### Data collection of quantitative study

**Fig. 2:**
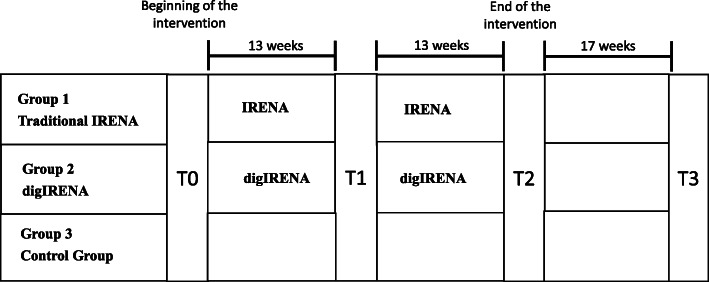
Design of the quantitative study part

The data collection started in January 2020. Due to the pandemic, the rehabilitation centers did not receive any patients from mid-March until about mid-May. To increase the number of recruited patients, two more rehabilitation centers joined the project in August 2020 (initially there were only three centers). The recruitment of the patients is planned to finish in October 2021. In total, the data collection includes four measurement occasions. The baseline measurement takes place at the rehabilitation center before the start of the intervention, the first follow-up measurement 13 weeks after the baseline, the second follow-up 26 weeks after the baseline, and the third follow-up 43 weeks after the baseline (see Fig. 2). The first three measurement occasions represent the development of the outcome variables, approximately, before, during and at the end of the intervention. Due to the limited duration of the study, the last follow-up measurement is intended to illustrate the sustainability of developments over the next 4 months. For the measurements 2–4, the questionnaires are directly sent to the patients, in accordance to their preference via post or e-mail. A reminder is sent in case the patients have not returned the questionnaires after 2 weeks.

#### Measures

The attendance, work ability, subjective health, and motivation will be measured to compare the development of the three groups (i.e., web-based aftercare, traditional face-to-face aftercare, control group). While the work ability, subjective health, and motivation are assessed at every measurement occasion, the attendance is not assessed at the first measurement occasion. Furthermore, attendance and motivation are only assessed in the web-based aftercare and the traditional face-to-face aftercare groups.

#### Attendance

This part of the questionnaire was developed for this study. Patients in the traditional face-to-face aftercare group and the web-based aftercare group are asked (a) how often they participated in their program and (b) how many minutes within a week on average they trained with their program. Furthermore, patients of all three groups will be asked about their average weekly participation in other sport activities.

#### Work ability

The German version of the Work Ability Index (WAI) as an internationally established instrument is used to measure the work ability of the patients [26]. The WAI consists of ten questions with categorical and continuous response formats, resulting in a score from a minimum of 7 to a maximum of 49. This score indicates the current assessment of one’s own work ability to deal with the work demands. The WAI has been shown to have an acceptable Cronbach᾽s α = 0.78 [27] and to predict an early work termination and long-term work disability [18, 19].

#### Subjective health

The German version of the SF-12 is used to measure the subjective health [28]. The SF-12 consists of 12 items with dichotomous and continuous response formats, which comprise the two dimensions physical and mental health. Studies have shown good Cronbach᾽s α = 0.83 for physical health and α = 0.87 for mental health [28]. Moreover, the SF-12 can be used independent of the current health status, and the questions have been deemed as understandable and relevant [29].

#### Motivation

The Behavioural Regulation Exercise Questionnaire-2 [BREQ-2 [30]], translated into German, is used to measure motivation. The questionnaire consists of 19 items with a five-point Likert scale, measuring five dimensions of motivation: intrinsic motivation, identified regulation, introjected regulation, extrinsic motivation, and amotivation. The reliability has been shown to range from acceptable to good for the different dimensions: intrinsic motivation (*α* = 0.88), identified regulation (*α* = 0.83), introjected regulation (*α* = 0.77), extrinsic motivation (*α* = 0.77), and amotivation [*α* = 0.60; 30]. In addition, the subscales show inter-correlations in line with theoretical considerations, and more autonomous types of motivation are associated with a higher probability of health behaviours [31].

#### Data analysis of quantitative data

Data are entered by research assistants and checked for accuracy by an independent research assistant. All personal information is pseudo-anonymized. Missing data are treated using either the multiple imputation or the full-information maximum likelihood approach. A first analysis will include all patients who intended to participate in the interventions and a second study will include only those patients who actually completed the interventions. A multivariate analysis of variance with repeated measurements will be used to analyze the data. Additionally, the development of work ability and subjective health will be analyzed by latent growth curve models [32]. These models allow the analysis of linear and nonlinear developments over time. A mediation analysis will be conducted to test whether motivation mediates the effects of the intervention on the attendance rate. The effects of age and gender will be controlled.

### Qualitative study

Parallel to the quantitative study, a qualitative study using interviews will be conducted to examine the applicability, acceptance and usability of the web-based aftercare program.

#### Data collection of qualitative study

Semi-structured interviews will be conducted with 13 therapists and 15 patients using the interview guidelines developed in accordance with the criteria of qualitative research (Tong, Sainsbury, & Craig, 2007). The content of the interview guidelines for both patients and therapists target applicability, acceptance, and usability of the web-based aftercare program. Particularly, the questions are formulated to assess the general experience with the program, its evaluations, and suggestions for improvement. It is intended to include patients who are still actively participating in the web-based aftercare program as well as patients who had dropped out of this program. The interviews will be conducted via Skype or over the phone due to the current Covid-19 pandemic.

#### Data analysis of qualitative data

For data transcription and data analysis, the use of the software MaxQDA is planned, which is explicitly designed to support transcriptions and analyses of qualitative data.

In order to specify the questions about the applicability, acceptance and usability of the web-based aftercare program, the data analysis is based on the Qualitative Content Analysis [33]. The categories will be developed as a combination of inductive and deductive category formation. As a result, strategies for improving the applicability, acceptability and usability of the web-based aftercare program will be developed. To ensure the rigour of the qualitative study, all steps are carried out by two independent researchers, implying an investigator triangulation [34]. In addition, there will be meetings with the other researchers of the team who will act as critical friends helping to reflect on alternative interpretations of the data [35].

## Discussion

The purposes of this study are 1) to assess the efficacy of a web-based platform used in the aftercare of a rehabilitation stay whilst looking at 1a) attendance, 1b) work ability, 1c) subjective health and 1d) motivation as well as 2) to gain deeper insights into how a web-based platform is perceived by therapists and patients whilst looking at 2a) applicability, 2b) acceptance and 2c) usability.

Due to an increased proportion of aging population in Germany, web-based rehabilitation aftercare could be an effective supplement to the traditional face-to-face rehabilitation aftercare. It has the potential to reach those people who cannot easily reach and not have access to traditional face-to-face rehabilitation aftercare. As traditional face-to-face rehabilitation aftercare suffers from low level of usage due to inflexible offers in terms of time and long distances to rehabilitation centers, web-based aftercare could help tackle some of these problems.

This study has several strengths and limitations. A strength of this study is the design as a partially randomized control trial, which enables us to draw strong conclusions about the effectiveness of the intervention. Additionally, the intended sample of the study is large enough to find even small effects. Furthermore, the longitudinal structure of the study enables us to examine development trajectories in the outcome variables. Finally, we use reliable and valid measures of the outcome variables, which have been tested in a vast amount of studies.

A limitation is that the corona pandemic partially interrupted the course of the study so that we lost a part of the participants due to this interruption. Therefore, we requested to include additional clinics and to prolong the duration of the study to reach the aimed number of participants. It is possible that the interruption could influence the results of the study.

### Trial status

The trial has been open for enrolment of participants between January 2020 and October 2021. The planned final assessment of the follow-up would be in July 2021. Due to the interruption caused by the corona pandemic, we requested a prolongation of the study for 1 year.

## Supplementary Information

**Additional file 1.** Questionnaire for patients in the IRENA-group.

**Additional file 2.** Questionnaire for patients in the digIRENA-group.

**Additional file 3.** Questionnaire for patients in the control group.

## Data Availability

The datasets used and/or analysed during the current study are available from the corresponding author on reasonable request.

## Abbreviations

SF-12: Short Form 12
WAI: Working Ability Index
BREQ-2: Behavioural Regulation Exercise Questionnaire-2
DRKS: German Register of Clinical Studies
IRENA: Intensivierte Rehabilitationsnachsorge (Intensified Rehabilitation Aftercare)
digIRENA: Digitalisierte IRENA (digitized Intensified Rehabilitation Aftercare)
ANOVA: Analysis of Variance

## References

[1] Bongaarts J (2009). Human population growth and the demographic transition. Philos Trans R Soc Lond B Biol Sci.

[2] Delivorias A, Sabbati G (2015). EU demographic indicators: situation, trends and potential challenges.

[3] England K, Azzopardi-Muscat N (2017). Demographic trends and public health in Europe. Eur J Public Health.

[4] Fuchs R, Goehner W, Seelig H (2011). Long-term effects of a psychological group intervention on physical exercise and health: the MoVo concept. J Phys Act Health.

[5] Fleig L, Pomp S, Schwarzer R, Lippke S (2013). Promoting exercise maintenance: how interventions with booster sessions improve long-term rehabilitation outcomes. Rehabil Psychol.

[6] DRV (2016). Die medizinische und berufliche Rehabilitation der Rentenversicherung im Licht der Statistik.

[7] Atasoy H, Greenwood BN, McCullough JS (2019). The digitization of patient care: a review of the effects of electronic health records on health care quality and utilization. Annu Rev Public Health.

[8] Agostini M, Moja L, Banzi R, Pistotti V, Tonin P, Venneri A, Turolla A (2015). Telerehabilitation and recovery of motor function: a systematic review and meta-analysis. J Telemed Telecare.

[9] Haluza D, Jungwirth D (2015). ICT and the future of health care: aspects of health promotion. Int J Med Inf.

[10] Lamprecht J, Behrens J, Mau W, Schubert M (2011). Das intensivierte rehabilitationsnachsorgeprogramm (irena) der deutschen rentenversicherung bund–berufsbegleitende inanspruchnahme und veränderungen berufsbezogener parameter. Die Rehabilitation.

[11] Frederix I, Vanhees L, Dendale P, Goetschalckx K (2015). A review of telerehabilitation for cardiac patients. J Telemed Telecare.

[12] Hailey D, Roine R, Ohinmaa A, Dennett L (2013). The status of telerehabilitation in neurological applications. J Telemed Telecare.

[13] Pastora-Bernal JM, Martín-Valero R, Barón-López FJ, Estebanez-Pérez MJ (2017). Evidence of benefit of telerehabitation after orthopedic surgery: a systematic review. J Med Internet Res.

[14] Elbert NJ, van Os-Medendorp H, van Renselaar W, Ekeland AG, Hakkaart-van Roijen L, Raat H, Nijsten TE, Pasmans SG (2014). Effectiveness and cost-effectiveness of ehealth interventions in somatic diseases: a systematic review of systematic reviews and meta-analyses. J Med Internet Res.

[15] Shenoy MP, Shenoy PD (2018). Identifying the challenges and cost-effectiveness of Telerehabilitation: a narrative review. J Clin Diagn Res.

[16] Rifkin J (1998). The end of work: the decline of the global labor force and the dawn of the post-market era. J Leis Res.

[17] Ilmarinen J (2009). Work ability—a comprehensive concept for occupational health research and prevention. Scand J Work Environ Health.

[18] Burdorf A, Frings-Dresen MHW, van Duivenbooden C, Elders LAM (2005). Development of a decision model to identify workers at risk of long-term disability in the construction industry. Scand J Work Environ Health.

[19] Salonen P, Arola H, Nygård CH, Huhtala H, Koivisto AM (2003). Factors associated with premature departure from working life among ageing food industry employees. Occup Med (Lond).

[20] Bullinger M. Assessment of health related quality of life with the SF-36 Health Survey. Die Rehabilitation. 1996;35:17-27. 8975342

[21] van der Linde RM, Mavaddat N, Luben R, Brayne C, Simmons RK, Khaw KT, Kinmonth AL (2013). Self-rated health and cardiovascular disease incidence: results from a longitudinal population-based cohort in Norfolk, UK. PLoS One.

[22] Berger N, Van der Heyden J, Van Oyen H (2015). The global activity limitation indicator and self-rated health: two complementary predictors of mortality. Arch Public Health.

[23] Teixeira PJ, Carraça EV, Markland D, Silva MN, Ryan RM (2012). Exercise, physical activity, and self-determination theory: a systematic review. Int J Behav Nutr Phys Act.

[24] IRENA – Intensivierte Rehabilitationsnachsorge [https://www.deutsche-rentenversicherung.de/DRV/DE/Reha/Reha-Nachsorge/IRENA/irena_node.html].

[25] Finne E, Englert C, Jekauc D (2019). On the importance of self-control strength for regular physical activity. Psychol Sport Exerc.

[26] Hasselhorn H-M, Freude G (2007). Der Work-ability-Index: Ein Leitfaden.

[27] Martus P, Freude G, Rose U, Seibt R, Jakob O (2011). Arbeits-und gesundheitsbezogene Determinanten von Vitalität und Arbeitsfähigkeit.

[28] Morfeld M, Kirchberger I, Bullinger M (2011). SF-36 Fragebogen zum Gesundheitszustand: Deutsche Version des Short Form-36 Health Survey.

[29] Bullinger M, Kirchberger I (1998). SF-36: Fragebogen zum Gesundheitszustand; Handanweisung.

[30] Markland D, Tobin V (2004). A modification to the behavioural regulation in exercise questionnaire to include an assessment of amotivation. J Sport Exer Psychol.

[31] Mahony R, Blake C, Matthews J, O’Donnoghue G, Cunningham C. Physical activity levels and self-determined motivation among future healthcare professionals: utility of the behavioral regulation in exercise questionnaire (BREQ-2). Physiotherapy Theory and Practice. 2019;35:884–90. 10.1080/09593985.2018.1457112 29659306

[32] Curran PJ, Muthén BO (1999). The application of latent curve analysis to testing developmental theories in intervention research. Am J Community Psychol.

[33] Mayring P, Fenzl T (2014). Qualitative inhaltsanalyse. Handbuch Methoden der empirischen Sozialforschung.

[34] Carter N, Bryant-Lukosius D, DiCenso A, Blythe J, Neville AJ (2014). The use of triangulation in qualitative research. Oncol Nurs Forum.

[35] Smith B, McGannon KR (2018). Developing rigor in qualitative research: problems and opportunities within sport and exercise psychology. Int Rev Sport Exerc Psychol.

