# Supplementary material for: Effects of a web-based rehabilitation aftercare on subjective health, work ability and motivation: a partially randomized controlled trial
Source: BMC Musculoskelet Disord. 2021 Apr 19;22:366. doi: 10.1186/s12891-021-04239-z (PMC8054846; doi:10.1186/s12891-021-04239-z)
Supplement: Supplementary file 2 — Additional file 2. Questionnaire for patients in the digIRENA-group. [file 12891_2021_4239_MOESM2_ESM.docx]

**Questionnaire for patients in the digIRENA-group**

After your discharge from the rehabilitation clinic, did you participate in a session with CASPAR? no □ yes □

if yes, please tell us the **number of weeks** you participated in at least one CASPAR session.
 (please provide an estimate if you cannot remember exactly)

__________________

Please also indicate how many minutes on average you have exercised with CASPAR **per week.**
(please provide an estimate if you cannot remember exactly)

|___________|___________|___________|___________|___________|___________|

1. min 30 60 90 120 150 180 min
2. After your discharge from the rehabilitation clinic, did you participate additionally in other sports/physical activities (e.g., rehabilitation sports, gym, company sports, club sports)?

no □ yes □

if yes,

… what type of activity(ies)?

_________________________________________

Please also indicate how many minutes you participate in these activities on average **per week.**

(please provide an estimate if you cannot remember exactly)

|___________|___________|___________|___________|___________|___________|

0 min 30 60 90 120 150 180 min
